# Severe varicella-zoster virus meningoencephalomyelitis coexisting with visceral disseminated varicella-zoster virus infection in a patient with lupus nephritis: A case report

**DOI:** 10.1097/MD.0000000000033459

**Published:** 2022-04-07

**Authors:** Tian Tao, Jun Chen, Kunlan Long, Lijia Zhi, Song Zhang, Shuqin Liu, Yuexian Ma, Hong Yan, Lizeyu Lv, Yue Xu, Ling Wu, Liangbin Zhao, Peiyang Gao

**Affiliations:** a Department of Nephrology, Hospital of Chengdu University of Traditional Chinese Medicine, Chengdu, Sichuan, China; b Department of Critical Medicine, Hospital of Chengdu University of Traditional Chinese Medicine, Chengdu, Sichuan, China; c Department of Radiology, Hospital of Chengdu University of Traditional Chinese Medicine, Chengdu, Sichuan, China.

**Keywords:** lupus nephritis, meningoencephalomyelitis, metagenomic next-generation sequencing, varicella-zoster virus, visceral disseminated varicella-zoster virus infection

## Abstract

Meningoencephalomyelitis and visceral dissemination infection are rare but life-threatening complications of either the primary infection or reactivation of varicella-zoster virus (VZV) in immunocompromised patients. To date, few studies have reported the co-existence of VZV meningoencephalomyelitis and the visceral dissemination of VZV infection.

**Patient concerns:**

A 23-year-old male was diagnosed with lupus nephritis class III and was being treated with oral prednisone and tacrolimus. The patient exhibited herpes zoster 21-day after the initiation of therapy and experienced unbearable abdominal pain and generalized seizures 11 days after the onset of a zoster rash. Magnetic resonance imaging showed progressive lesions in the cerebrum, brainstem, and cerebellum, as well as meningeal thickening and thoracic myelitis. Computed tomography showed pulmonary interstitial infiltration, partial intestinal dilatation, and effusion. Metagenomic next-generation sequencing revealed 198,269 and 152,222 VZV-specific reads in the cerebrospinal fluid and bronchoalveolar lavage fluid, respectively.

**Diagnoses:**

Based on the clinical and genetic findings, this patient was finally diagnosed with VZV meningoencephalomyelitis and visceral disseminated VZV infection.

**Interventions:**

The patient received intravenous acyclovir (0.5 g every 8 hours) combined with plasma exchange and intravenous immunoglobulin. Treatment against secondary bacterial and fungal infections, organ support therapy and rehabilitation training were given simultaneously.

**Outcome:**

The patient’s peripheral muscle strength did not improve and repeated metagenomic next-generation sequencing showed the persistence of VZV-specific reads in the cerebrospinal fluid. The patient finally abandoned therapy due to financial constraints at the 1-month follow-up.

**Lessons:**

Patients with autoimmune diseases receiving immunosuppressive therapy should be warned about the possibility of developing serious neurological infections and visceral disseminated VZV infections as side effects. Early diagnosis and the early initiation of intravenous acyclovir therapy are important for such cases.

## 1. Introduction

Varicella-zoster virus (VZV), also known as human herpesvirus 3, is a neurotropic human herpesvirus that causes varicella during primary infection and herpes zoster (HZ) during the reactivation of latent VZV in the ganglia.^[1,2]^ Previous research has shown that HZ events occur at a rate several times higher in immunocompromised patients as compared to the general population.^[3,4]^ In addition, severe complications; including VZV central nervous system (CNS) infection and visceral disseminated VZV infection; are more frequent in immunocompromised patients.^[5,6]^

CNS infection caused by VZV typically manifests as vasculopathy, encephalitis, cerebellitis, meningitis, and myelitis, with or without rash.^[7,8]^ Relatively few studies have reported the co-existence of meningitis, encephalitis, and myelitis in such cases. Patients suffering from meningoencephalomyelitis usually have a worse prognosis with a multitude of neurological sequelae.^[9–11]^ Visceral disseminated VZV infection is another rare but life-threatening complication of VZV infection.^[12,13]^ Visceral organ involvement may present as severe abdominal pain, hepatitis, pancreatitis, pneumonia, and other symptoms.^[6,14–16]^ Herein, we report a rare case of severe VZV meningoencephalomyelitis with visceral disseminated VZV infection in a patient with lupus nephritis (LN) by metagenomic next-generation sequencing (mNGS).

## 2. Case presentation

**Figure 1.**
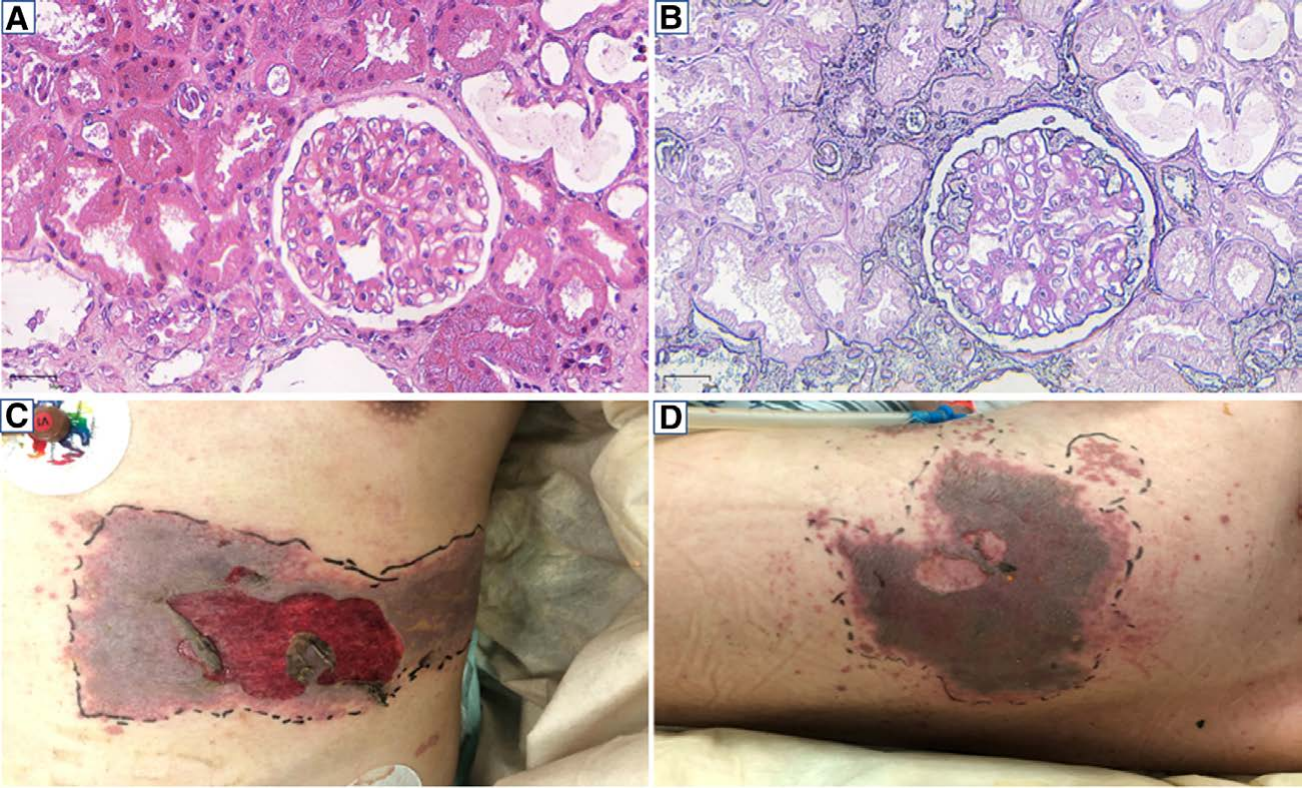
Renal biopsy findings and skin lesions. (A) The glomeruli showed mild to moderate hypercellularity and matrix expansion (hematoxylin-eosin staining × 200), (B) the glomeruli showed immune complexes deposited in mesangial and subendothelial regions (periodic acid-Schiff stain × 200), (C) clustered vesicular rash with ruptures apparent on the left hypochondrium (the patient was in the supine position), and (D) clustered vesicular rash with ruptures apparent on the left back (the patient was in the right lateral position).

A 23-year-old male was first admitted to the hospital on April 26th, 2022, due to having developed a malar rash and experiencing swelling of the lower extremities for 1 week. Physical examination revealed facial butterfly erythema, enlarged lymph nodes, periungual vasculitis-like rash, bilateral lower extremity edema, and normal blood pressure. Auxiliary examination (Table 1) showed a serum creatinine concentration of 1.74 mg/dL, serum albumin concentration of 21.8 g/L, urine protein content of 3.91 g/24 hours, complement 3 level of 0.48 g/L, complement 4 level of 0.16 g/L, thyroid stimulating hormone level of 6.508 miu/L, and free triiodothyronine concentration of 3.43 pmol/L. The patient was positive for antinuclear antibodies 1:3200 and was also strongly positive for anti-Smith antibodies, anti-ribonucleoprotein antibodies, and anti-ribosomal P protein antibodies. Further investigations revealed negative serology results for hepatitis B, C, and human immunodeficiency virus. The chest computed tomography (CT) scan showed regional pulmonary fibrosis. Renal biopsy revealed the presence of glomerular hypercellularity and mesangial proliferation with accompanying thickening of the glomerular basement membrane. Exfoliated renal tubular epithelial cells and dilated tubular lumen were also observed (Fig. 1A and B). Immunofluorescence microscopy showed deposits of immunoglobulin G, IgM, IgA, C3, and C1q in the mesangial area and capillary wall. Electron microscopy revealed electron-dense deposits in the mesangial and subepithelial space. The patient was diagnosed with systemic lupus erythematosus (SLE) and LN class III according to the International Society of Nephrology and Society of Renal Pathology classification criteria. Treatment was initiated with prednisone at 40 mg/day, tacrolimus at 1 mg twice daily, and hydroxychloroquine at 200 mg twice daily. Levothyroxine was administered to treat the hypothyroidism. One week after the initiation of the induction therapy, his serum creatinine decreased to 0.79 mg/dL and his urine protein content decreased to 0.03 g/24 hours. Following this, the patient was discharged.

**Table 1:** Laboratory data on admission.

Parameter	First admission	Second admission	Standard value
Hemoglobin	144 g/L	136 g/L	130–175 g/L
White blood cell count	4.82 × 109/L	8.15 × 109/L	3.5–9.5 × 109/L
Platelet count	173 × 109/L	144 × 109/L	100–300 × 109/L
Serum creatinine	1.74 mg/dL	0.93 mg/dL	0.65–1.10 mg/dL
Serum albumin	21.8 g/L	38.3 g/L	40–55 g/L
Proteinuria	3.91 g/24 h	-	≤0.15g/24 h
semiquantitative urinary protein	4+	negative	negative
Complement 3	0.48 g/L	0.65 g/L	0.79–1.52 g/L
Complement 4	0.16 g/L	0.15 g/L	0.16–0.38 g/L
Thyroid stimulating hormone	6.51 miu/L	0.50 miu/L	0.27–4.2 miu/L
Free triiodothyronine	3.43 pmol/L	0.93 pmol/L	1.3–3.1pmol/L
Antinuclear antibodie	1:3200	1:3200	<1:100
CD4+/CD8 + T cell ratio	-	0.2	1.5–2.5

**Figure 2.**
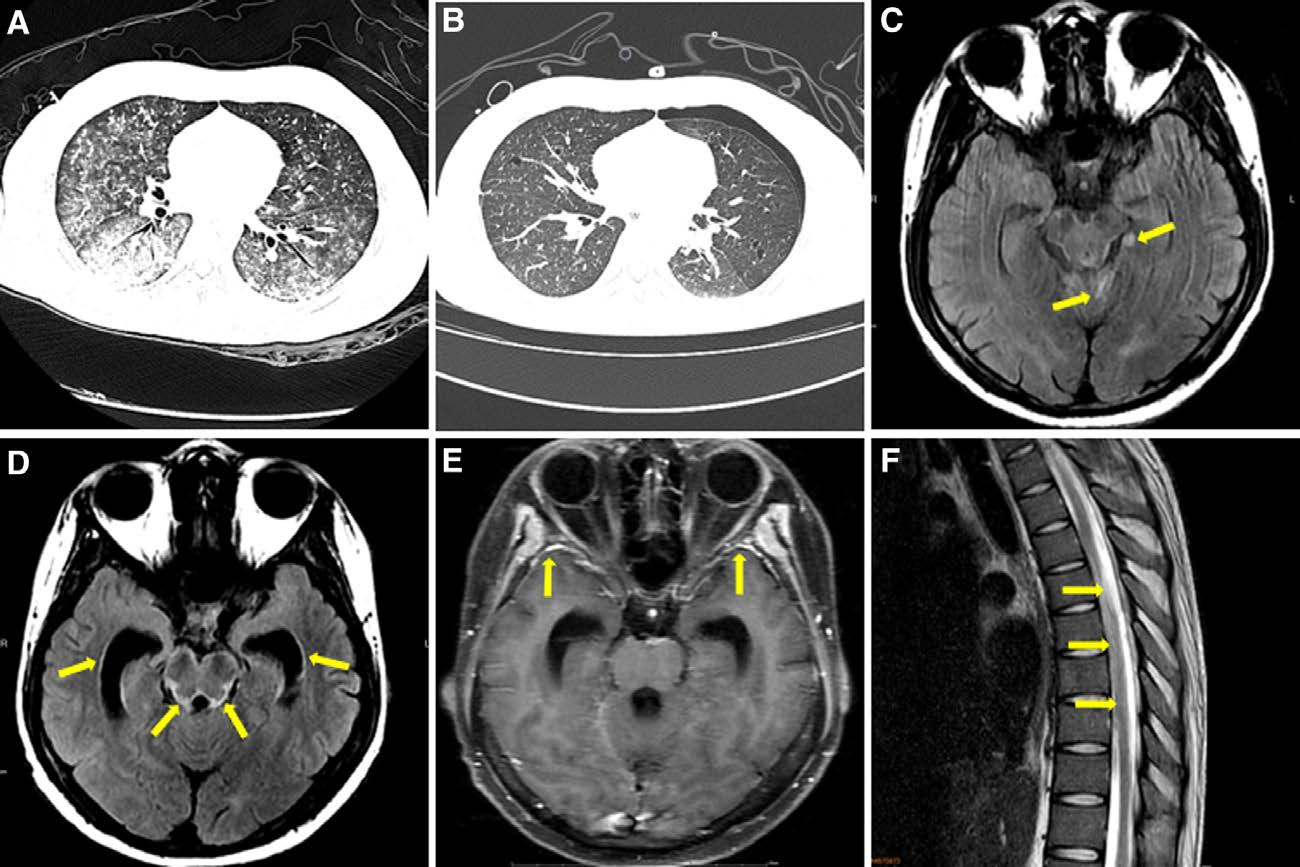
Imaging examination of the patient. (A) The chest computed tomography (CT) scan showed bilateral pulmonary interstitial infiltrates on 1 June 2022, (B) the follow-up chest CT scan showed improvement in the pulmonary lesions on 25 July 2022, (C) the T2WI FLAIR showed high signals in the left parahippocampal gyrus and cerebellar vermis on 1 June 2022 (yellow arrow), (D) the T2WI FLAIR showed widening of the ventricle on 21 July 2022 (yellow arrow), (E) the contrast-enhanced T1WI showed meningeal enhancement on 21 July 2022 (yellow arrow), and (F) the T2WI FLAIR showed longitudinally extensive spinal cord lesions on 21 July 2022 (yellow arrow).

The patient was readmitted to the hospital on May 28th, 2022, presenting with a painful rash with blisters on the left thoracodorsal area for 1 week. Physical examination conducted on the second admission showed a large band-like rash with vesicles in the left thoracic and lumbar dermatomes (Fig. 1C and D). The visual analog scale score was 6. No edema or new butterfly erythema was found. Laboratory tests showed a serum creatinine concentration of 0.93 mg/dL, serum albumin concentration of 38.3 g/L, complement 3 level of 0.65 g/L, and complement 4 level of 0.15 g/L. Urine protein was negative. The total T lymphocyte, CD4 + T cell, and CD8 + T cell counts were 699 cells/uL, 115 cells/uL, and 572 cells/uL, respectively. In addition, the CD4+/CD8 + T cell ratio decreased to 0.2. The patient presented with unbearable abdominal pain after admission and developed generalized seizures on June 1st, 2022. After tracheal intubation, he was transferred to the intensive care unit for further treatment. Chest CT showed bilateral pulmonary interstitial infiltrates (Fig. 2A). The results of brain magnetic resonance imaging (MRI) showed abnormal long *T*1 and long *T*2 signals in the left parahippocampal gyrus and cerebellar vermis (Fig. 2C). A lumbar puncture was then performed, which detected an elevated intracranial pressure of 270 mm H_2_O. The cerebrospinal fluid (CSF) analysis showed a white blood cell count of 78 × 10^6^ cells/L (84.6% neutrophils), glucose level of 9.5 mmol/L, and protein level of 5.03 g/L. No significant pathogens were isolated in the CSF culture. The CSF sample and bronchoalveolar lavage fluid (BALF) sample were sent for mNGS analysis to identify the responsible pathogen on June 1st, 2022. Written informed consent was provided by the patient’s parents.

**Figure 3.**
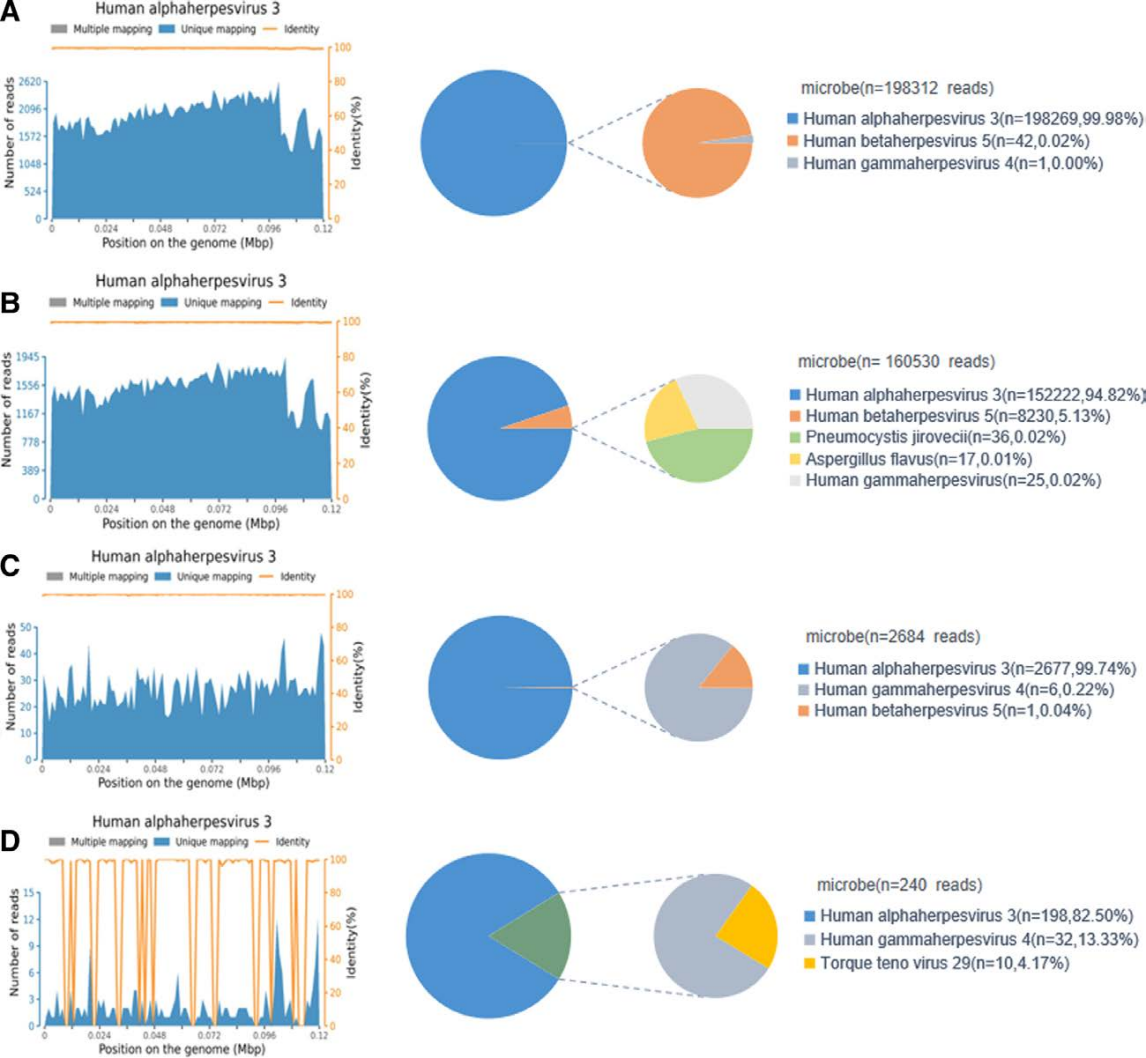
Metagenomic next-generation sequencing (mNGS) results of the patient. (A) The mNGS results for pathogen identification in cerebrospinal fluid (CSF) on 2 June 2022, (B) the mNGS results of bronchoalveolar lavage fluid (BALF) on 2 June 2022, (C) the mNGS results of CSF on 17 June 2022, and (D) the mNGS results of CSF on 8 July 2022.

The first CSF mNGS result showed 198,269 specific reads uniquely matched to the VZV genome with a coverage of 99.93%. In addition, trace cytomegalovirus (CMV) sequences were detected in the CSF sample (42 sequence reads) (Fig. 3A). The mNGS result for the BALF sample showed 152,222 distinct VZV reads with a coverage of 99.94%. A total of 8230 CMV-specific reads were also identified in the BALF sample with a coverage of 77.01%. Additionally, traces of *Pneumocystis jirovecii* and *Aspergillus flavus* sequences were detected in the BALF sample (36 and 17 sequence reads, respectively) (Fig. 3B). The patient then received intravenous acyclovir (0.5 g every 8 hours) for 16 days, ganciclovir (0.25 g every 12 hours) for 14 days, intravenous immunoglobulin once per day for 3 days, and plasma exchange once per day for 4 days. The patient also received levothyroxine, methylprednisolone, cyclophosphamide, antibacterial drugs, and antifungal drugs.

Although the patient gradually recovered consciousness after treatment, his respiratory function and peripheral muscle strength were markedly diminished (the upper and lower limbs were grade 2/5 and 0/5, respectively). Furthermore, the patient still complained of experiencing abdominal pain, while abdominal CT showed partial intestinal dilatation and intestinal effusion. A lumbar puncture was re-performed to observe cytologic and protein changes in the CSF and to allow for mNGS testing on June 17th, 2022. The analysis of the CSF showed a decreased number of white blood cells and decreased protein levels (a white blood cells count of 47.6 × 10^6^ cells/L and a protein concentration of 1.01 g/L). The results obtained by mNGS conducted on the CSF sample detected 2677 unique deoxyribonucleic acid (DNA) reads of VZV and had a genomic coverage of 64.34%. Only 1 CMV-specific read was detected in the CSF sample (Fig. 3C). The patient subsequently received an additional course of acyclovir (0.5 g every 8 hours) for 21 days, which was accompanied by antibacterial treatment, antifungal treatment, and rehabilitative therapy.

**Figure 4.**
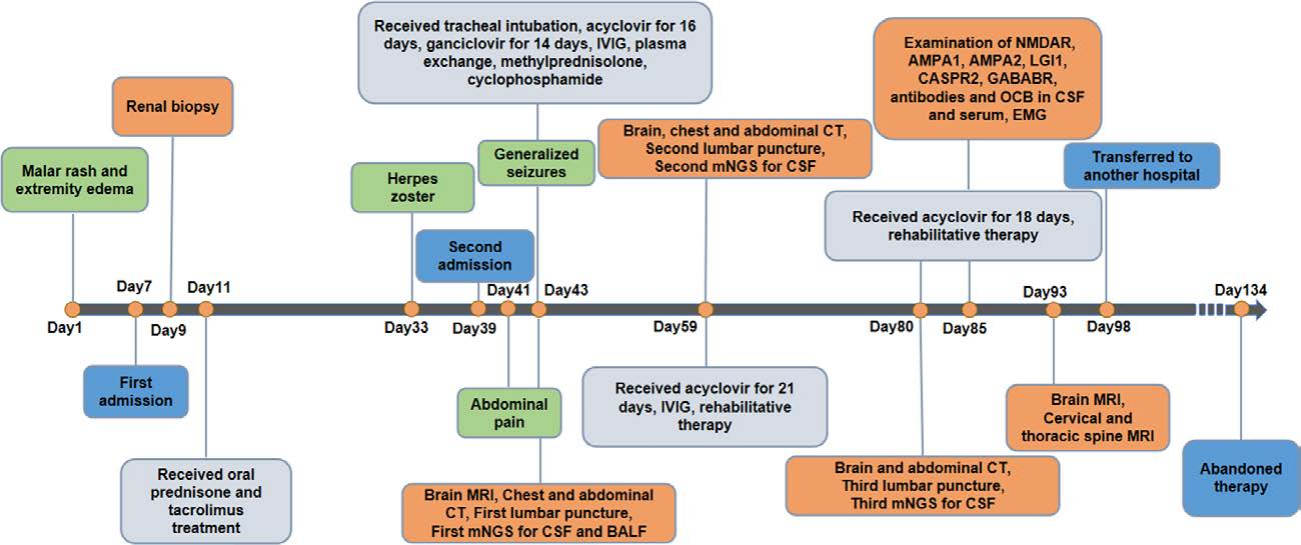
Timeline of the clinical course and duration of treatment.

Under uninterrupted antiviral therapy and rehabilitative treatment, the patient partially restored their respiratory capacity. However, their peripheral muscle weakness did not improve significantly. Electromyography showed decreased motor and sensory nerve conduction velocities. Specific autoantibodies for autoimmune encephalitis (NMDAR, AMPA1, AMPA2, LGI1, CASPR2, and GABABR) were negative both in the serum and CSF. The detection of identical oligoclonal immunoglobulin G bands in the serum and CSF confirmed the disruption of the blood-CSF barrier. A third lumbar puncture was performed on July 8th, 2022. The CSF analysis showed that the white blood cell count decreased to 8 × 10^6^ cells/L and the protein level decreased to 0.55 g/L. Furthermore, the mNGS results showed that the unique VZV readings count decreased to 198 and had a coverage of 7.19% (Fig. 3D). As the patient still tested positive for VZV infection, acyclovir treatment and supportive treatment were continued. Although the follow-up chest CT scan showed improvement in the pulmonary lesions (Fig. 2B), the repeated head MRI showed increased abnormal *T*1 and *T*2 signals in the cerebrum, brainstem, and cerebellum, as well as marked meningeal thickening and ventricular widening on July 21^st^, 2022 (Fig. 2D–E). Moreover, cervical and thoracic spine MRI showed extensive swelling and focal atrophy of the thoracic spinal cord (Fig. 2F). The patient was finally diagnosed with VZV meningoencephalomyelitis and visceral disseminated VZV infection. He was subsequently transferred to another hospital to receive further treatment on July 26th, 2022. The patient ultimately abandoned therapy due to financial constraints at the 1-month follow-up. The clinical course and duration of treatment are summarized in Figure 4.

## 3. Discussion and conclusion

VZV is a highly infectious herpesvirus that causes varicella during primary infection and HZ during reactivation.^[1,17]^ Cell-mediated immunity plays an important role in the prevention of VZV reactivation in adults.^[18]^ Patients affected by autoimmune diseases like SLE have a significantly increased risk of VZV reactivation. Treatment with glucocorticoids and immunosuppressants may further increase this risk.^[4,19]^ Moreover, immunocompromised individuals are more likely to present rare but potentially lethal manifestations, such as meningoencephalomyelitis and disseminated visceral involvement.^[20]^ VZV meningoencephalomyelitis has been reported to be associated with the direct viral invasion of the spinal cord and retrograde viral transport to the brain.^[21,22]^ Meanwhile, the spread of VZV from the dorsal root ganglion toward the viscera may be related to visceral disseminated VZV infection.^[12,23]^ In the present case, the patient received oral prednisone and tacrolimus from the time of being diagnosed with LN class III. To avoid relapse, the doses of the steroids and immunosuppressants were not reduced immediately when the patient achieved complete remission of proteinuria within a week. Although the time interval between the initiation of immunosuppressive therapy and the onset of the zoster rash was only 21 days, the induction therapy caused a marked decrease in the lymphocyte counts of this patient. Notably, lymphocytopenia (<1000 cells/μL) is considered an independent risk factor for the development of severe infections in patients with SLE.^[24]^

Our patient originally suffered from a zoster rash 1 week before the onset of abdominal pain and generalized seizures. The association between the neurological symptoms and HZ was properly established by mNGS to identify specific VZV sequences in the CSF. Although trace CMV-specific reads were also detected in the CSF, they almost turned negative after 14 days of ganciclovir treatment. In contrast, the patient had persistent VZV-specific sequences in the CSF along with progressively intracranial lesions and thoracic spine myelitis on MRI. Therefore, the diagnosis of meningoencephalomyelitis caused by VZV was considered. Moreover, the large number of VZV-specific reads detected by mNGS in the BALF provided crucial evidence for the disseminated seeding of the lung, leading to the inference that the unexplained abdominal pain was caused by disseminated VZV seeding the intestine. Studies have reported that high viral loads of VZV are associated with a higher incidence of severe intracranial lesions and more frequent neurological sequelae.^[25]^ Regrettably, the application of the quantitative polymerase chain reaction to VZV DNA in the CSF was not performed during the patient’s disease course. We only speculate that our patient may have had a high viral load of VZV in the CSF at the time of the first presentation, as there were nearly 2 hundred thousand VZV-specific reads detected by mNGS in the CSF. In addition, this number of VZV-specific reads decreased after continuous antiviral treatment, which was consistent with the changes observed in the biochemical indexes of the CSF after therapy.

Intravenous acyclovir has been widely accepted as the primary therapy for disseminated VZV infection and severe VZV CNS infection. The early use of antiviral drugs at the onset of initial symptoms is recommended to improve prognosis. The patient started antiviral therapy eleven days after the onset of the zoster rash, and this may be partly responsible for their poor prognosis. The optimum dose and duration of antiviral therapy remain unknown.^[26]^ Several studies have recommended intravenous acyclovir at a dose of 10 to 15 mg/kg 3 times daily for 14 to 21 days in such cases.^[27,28]^ It has also been suggested that treatment be discontinued when the CSF tests negative for VZV DNA.^[9]^ Although our patient was treated with intravenous acyclovir for 55 days in conjunction with plasma exchange and intravenous immunoglobulin, VZV-specific sequences were still detected in the CSF. The persistent presence of VZV-specific reads in the CSF may also be associated with the poor prognosis of this patient.

In conclusion, we have reported a rare case of VZV-induced severe meningoencephalomyelitis and disseminated visceral infection in a patient with LN treated with tacrolimus and high doses of glucocorticoids. Patients with autoimmune diseases receiving immunosuppressive therapy should be warned about the increased risk of develop VZV infection and reactivation. mNGS is essential for the early diagnosis of insidious neurological and visceral disseminated VZV infections in immunocompromised patients, and the early initiation of intravenous acyclovir therapy is important for such cases.

## Author contributions

**Conceptualization:** Tian Tao, Jun Chen.

**Data curation:** Kunlan Long, Lijia Zhi, Yuexian Ma, Hong Yan.

**Formal analysis:** Song Zhang, Shuqin Liu.

**Methodology:** Lizeyu LV, Yue Xu.

**Project administration:** Liangbin Zhao, Peiyang Gao.

**Software:** Ling Wu.

**Writing - original draft:** Tian Tao, Jun Chen.

**Writing - review & editing:** Liangbin Zhao, Peiyang Gao.
